# The Role of Fast Blood Glucose to Albumin Ratio in Predicting Gout Relief Among Patients With Acute Gout Attack

**DOI:** 10.1155/ije/2656529

**Published:** 2026-07-24

**Authors:** Wen Dai, Zebo Hu, Wenjian Mao, Jia Wu, Jun Hong

**Affiliations:** ^1^ Department of Clinical Laboratory, Jinling Hospital, Affiliated Hospital of Medical School, Nanjing University, Nanjing, China, nju.edu.cn, njmu.edu.cn; ^2^ Department of Critical Care Medicine, Jinling Hospital, Affiliated Hospital of Medical School, Nanjing University, Nanjing Jiangsu, 210002, China, nju.edu.cn

**Keywords:** acute gout attack, fast blood glucose to albumin ratio, gout relief, prediction model, uric acid

## Abstract

**Background:**

Early identification of patients with a high probability of gout relief is crucial for optimizing treatment strategies and improving patient outcomes. Thus, we aim to investigate the performance of some inflammatory and metabolic related indices and their combination in predicting gout relief.

**Methods:**

This is a retrospective analysis using data extracted from a single‐center database. The predictive performance of fast blood glucose to albumin ratio (FAR), fasting blood glucose to prealbumin ratio (FPR), C‐reactive protein to prealbumin ratio (CAR), and conventional indices were assessed by the area under the receiver operating characteristic curve. Multivariable logistic regression analysis (MLRA) was used to define the relationship between the best‐performing index and gout relief within 28 days of admission. The generalized additive model (GAM) and eXtreme Gradient Boosting (XGBoost) model were used to verify the conclusion.

**Results:**

From January 2022 to April 2023, 260 patients with acute gout attack were enrolled for further analysis. Patients were divided into training cohort (*n* = 182) and internal validation cohort (*n* = 78). FAR was chosen to be enrolled in the final model during Lasso regression variables’ selection procedure. In the MLRA model, FAR (OR [95 CI%]: 0.59 [0.36–0.96], *p* = 0.035) was found to be independently associated with gout relief. The GAM and XGBoost model yielded similar result. The predictive performance of the model with FAR was significantly better than that without FAR, as reflected by a higher C index (DeLong test *p* = 0.016).

**Conclusion:**

FAR at admission could be a novel and effective predictor for gout relief. The integration of FAR into a comprehensive prediction model represents a significant step forward in gout prognostication.

## 1. Introduction

Gout is a common form of inflammatory arthritis characterized by recurrent attacks of acute joint inflammation caused by the deposition of monosodium urate crystals [1]. While effective treatments like allopurinol exist, the management of gout remains suboptimal, with many patients experiencing persistent symptoms and complications [2–4]. Early identification of patients with a high probability of gout relief is crucial for optimizing treatment strategies and improving patient outcomes.

Traditional predictors of gout relief or frequent flares, such as serum uric acid (URIC) levels and the presence of tophi, have limitations in their predictive accuracy [5, 6]. Recent studies have explored novel biomarkers and ratios to enhance the prediction of disease outcomes in various conditions, including inflammatory and metabolic disorders [7–10]. Among these, ratios involving fast blood glucose (FBG), albumin (ALB), prealbumin (PA), and C‐reactive protein (CRP) have shown promise in assessing disease severity.

The FBG to albumin ratio (FAR) has been investigated as a potential marker of postoperative pneumonia in older adults with hip fractures [11]. The CRP to albumin ratio (CAR) has emerged as a valuable prognostic indicator in various inflammatory and malignant conditions [12, 13]. However, the potential of these novel indices in predicting gout relief remains unexplored.

Our study aims to investigate the performance of FAR and CAR in predicting gout relief, addressing a significant gap in the current literature. By evaluating these novel indices, we seek to develop a more comprehensive and accurate predictive model for gout relief, which may help guide personalized treatment strategies, and serve as valuable tools in clinical trials, aiding in patient stratification and the assessment of treatment efficacy.

## 2. Methods

### 2.1. Study Design and Participants

This study is a retrospective analysis using data from a single‐center database. All data were collected prospectively during hospitalization with the consent of the patients or their next of kin. Consecutive patients who met the following criteria were included: (1) with crystal proven gout and acute symptoms, (2) admitted within 3 days of the advent of symptoms, and (3) age between 18 and 80 years. Patients who met the following criteria were excluded: (1) in pregnancy/lactation, (2) had severe pre‐existing comorbidities, or (3) complete loss of laboratory indices and/or primary endpoint.

### 2.2. Data Collection

All the data required in this secondary analysis were extracted from a prospectively collected database. The establishment of the database was approved by the Review Committee of Jinling Hospital (No: 2025DZKY‐016‐02). The baseline characteristics include age, gender, times of gout attack within the past 12 months before admission, HLAB5801 gene sensitivity, and visual analog scale (VAS) score at admission. Laboratory indices included white blood cell (WBC), CRP, FBG, D‐dimer (DD), ALB, PA, erythrocyte sedimentation rate (ESR), and URIC. In addition, FBG to ALB ratio (FAR), FBG to PA ratio (FPR), and CRP to ALB ratio (CAR) were calculated.

### 2.3. Clinical Outcome

The primary outcome was gout relief within the first 28 days of admission. Gout relief was defined as the absence of acute or chronic inflammation symptoms caused by tissue deposits, including pain, joint damage, and functional disability, as previously described [14]. The selection of the 28‐day time window mainly refers to a previous randomized controlled trial [15]. Patients who were readmitted due to a new‐onset gout attack within 28 days were considered as not relief. Patients discharged from the hospital within 28 days were followed up through outpatient or telephone. Medicines (nonsteroidal anti‐inflammatory drugs, colchicine, and hormones) for gout arthritis management are used in accordance with the latest guidelines [16, 17]. We anticipated a 30% prevalence of gout alleviation within 28 days of hospitalization. According to the 10 Events Per Variable (10EPV) approach, a sample size of more than 250 might offer 75 events while allowing enrollment of a maximum of 7 variables.

### 2.4. Statistical Methodology

The Kolmogorov–Smirnov test was used to assess the normality of quantitative data. The continuous variables were tested by the Student′s *t*‐test (normally distributed) or Mann–Whitney *U* test (nonnormally distributed). The mean ± standard deviation (SD) values and median (interquartile range [IQR]) were used to describe continuous variables, whereas categorical data are expressed as frequencies and percentages. The comparison of categorical data between groups was performed using the chi‐square test or Fisher’s exact test.

We divided all patients of the study cohort into the training cohort and internal validation cohort in a 7:3 ratio randomly. We follow two rules to enroll the variables: (1) potential differences between groups with a *p* value less than 0.10 and (2) potentially relevant variables according to previous studies and clinical considerations. The associations between the composite indices like FAR and their component variables (glucose and ALB), as well as other inflammatory or metabolic indicators, were assess by the Spearman’s correlation coefficient, and the regression lines and heatmap for correlations were drawn. To establish a robust and accurate risk model, we subsequently carried out a least absolute shrinkage and selector operation (LASSO) analysis to filter out redundant factors through the glmnet package in R. The lambda value was screened through cross‐validation, and the model was constructed using the lambda‐min value. Collinearity was additionally tested to ensure the independence of each variable, by calculating the values of variance inflation factor (VIF). As a result, male, URIC, FAR, FPR, VAS score at admission, and gout frequencies during 12 months before admission were involved (Figure S1). Then, the multivariable logistic regression models with FAR or without FAR were used to evaluate the association between FAR and gout relief. In addition, we also introduced generalized additive model (GAM) and the eXtreme Gradient Boosting (XGBoost) model to verify that the conclusions do not rely on linear assumptions.

Predictive performance was assessed by the area under the receiver operating characteristic curve (AUC). The Youden’s Index of receiver operator characteristic (ROC) analysis was used to define the optimal cutoff point and corresponding sensitivity and specificity for each model to predict gout relief. The DeLong test is a nonparametric approach developed by Elizabeth R et al. [18]. DeLong et al. was then used to compare the differences in AUC. Calibration curves were used to evaluate the accuracy and consistency of the models. In addition, the association between FAR and gout relief were analyzed among different age stratum (> 50 or ≤ 50 years old). P for interaction was also reported.

Statistical tests were two‐sided, and *p* values < 0.05 were considered significant unless, otherwise, stated. All data processing was done in SPSS 27.0 software and R 4.4.3 software (R Foundation for Statistical Computing).

## 3. Results

### 3.1. Characteristics of the Study Population

**FIGURE 1:**
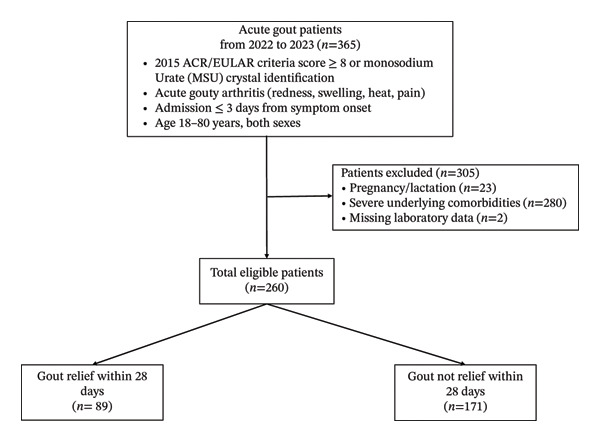
Flowchart.

**FIGURE 2:**
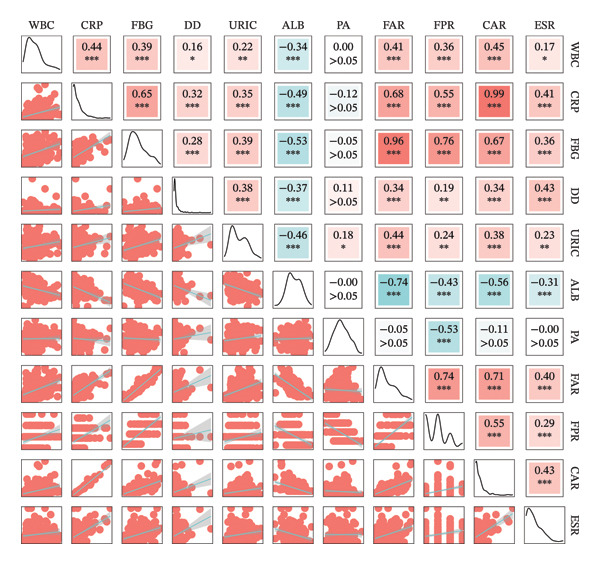
Correlations between each laboratory indices. WBC denotes white blood cell, CRP denotes C‐reactive protein, FBG denotes fast blood glucose, DD denotes D‐dimer, ALB denotes albumin, PA denotes prealbumin, FAR denotes FBG/ALB ratio, FPR denotes FBG/PA ratio, CAR denote CRP/FBG ratio, ESR denotes erythrocyte sedimentation rate, and URIC denotes uric acid.

From January 2022 to April 2023, 260 patients were enrolled in this analysis (Figure 1). Among them, 95% of the patients are male. Within 28 days of admission, 89/260 (34.2%) patients have gout relief. The demographic and baseline characteristics of the two groups in the training cohort and the internal validation cohort are shown in Table 1. In the training cohort, patients in the gout relief group have less frequencies of gout attack during the past 12 months before admission and lower VAS score at admission. For the laboratory indices, patients in the gout relief group had a lower level of WBC CRP, FBG, DD, FAR, FPR, CAR, ESR, and URIC and a higher level of ALB (all *p* < 0.05), compared to those in the nonrelief group. Correlations between each laboratory indices are shown in Figure 2. All the composite indices were significantly correlated with their components.

**TABLE 1:** Baseline and clinical characteristics of patients with gout relief.

	Overall (*n* = 260)	Training cohort (*n* = 182)			Internal validation cohort (*n* = 78)		
		Gout relief group (*n* = 89)	Nonrelief group (*n* = 171)	*p*	Gout relief group (*n* = 89)	Nonrelief group (*n* = 171)	*p*
Age	51 (38–63)	50 (40.5–62.5)	53 (37–66)	0.906	50.5 (39.5–60.5)	44.5 (37.5–60.0)	0.525
Sex, male, *n* (%)	247 (95.0)	56 (98.2)	118 (94.4)	0.438	29 (90.6)	44 (95.7)	0.373
History of gout attack within the past 12 months ≥ 3 times, *n* (%)	18 (23.1)	1 (1.8)	34 (27.2)	< 0.001	3 (9.4)	15 (32.6)	0.017
VAS at admission	2 (0–4)	0	3 (2–5)	< 0.001	0	3 (1–4)	< 0.001
HLAB5801 gene sensitivity, *n* (%)	31 (11.9)	8 (14.0)	16 (12.8)	0.335	5 (15.6)	2 (4.3)	0.087
Laboratory indices							
WBC, 10^9^/L	7.8 (5.8–9.8)	6.2 (5.2–8.2)	8.4 (6.3–10.2)	< 0.001	7.2 (5.6–8.9)	8.0 (6.3–10.9)	0.268
CRP, (mg/L)	14.1 (1.8–35.1)	1.8 (0.8–14.8)	23.1 (5.1–48.1)	< 0.001	4.2 (1.0–15.2)	22.1 (2.8–39.4)	0.002
FBG, mmol/L	6.0 (5.1–7.4)	5.6 (5.1–5.9)	6.7 (5.8–7.2)	< 0.001	4.6 (4.2–5.1)	6.8 (5.5–8.3)	< 0.001
DD, μg/mL	0.54 (0.25–1.22)	0.31 (0.17–0.56)	0.69 (0.32–1.59)	< 0.001	0.46 (0.23–0.77)	0.76 (0.26–1.54)	0.078
ALB, g/L	38.6 (34.9–42.6)	42.4 (39.5–45.3)	35.9 (33.8–39.9)	< 0.001	41.6 (39.5–43.0)	36.2 (34.2–40.2)	< 0.001
PA, g/L, mean ± SD	260.0 ± 60.3	251.0 ± 66.8	260.0 ± 58.3	0.411	268.8 ± 60.1	263.8 ± 58.1	0.716
FAR	0.10 (0.08–0.16)	0.09 (0.07–0.09)	0.14 (0.10–0.18)	< 0.001	0.06 (0.05–0.07)	0.13 (0.09–0.18)	< 0.001
FPR	0.02 (0.01–0.02)	0.01 (0.01–0.02)	0.02 (0.01–0.03)	< 0.001	0.01 (0.01–0.01)	0.02 (0.01–0.02)	< 0.001
CAR	0.35 (0.04–0.96)	0.04 (0.02–0.36)	0.65 (0.12–1.24)	< 0.001	0.10 (0.02–0.36)	0.61 (0.07–1.08)	0.002
ESR, mm/h	23.0 (8.0–48.5)	11 (5–31)	31 (16–56)	< 0.001	15.0 (6.0–56.8)	14.5 (8.0–43.3)	0.729
URIC, umol/L	377.0 (283.3–543.8)	270.0 (245.0–296.5)	492.0 (363.5–590.0)	< 0.001	271 (246–336.5)	524 (393–610.8)	< 0.001

*Note:* ALB denotes albumin, PA denotes prealbumin, CAR denote CRP/FBG ratio, and URIC denotes uric acid.

Abbreviations: VAS, visual analog scale; WBC, white blood cell; CRP, C‐reactive protein; FBG, fast blood glucose, DD, D‐Dimer; FAR, FBG/ALB ratio; FPR, FBG/PA ratio; ESR, erythrocyte sedimentation rate.

### 3.2. Factors Associated With Gout Relief

**FIGURE 3:**
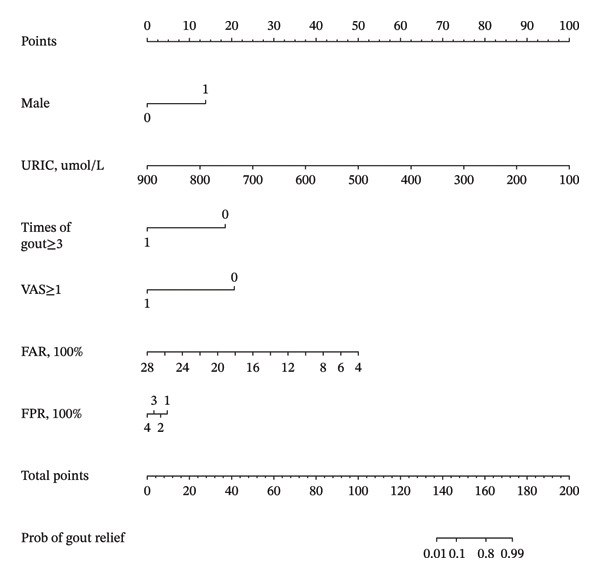
Nomogram for predicting gout relief. VAS denotes visual analog scale, FAR denotes FBG/ALB ratio, FPR denotes FBG/PA ratio, FBG denotes fast blood glucose, ALB denotes albumin, PA denotes prealbumin, and URIC denotes uric acid.

**FIGURE 4:**
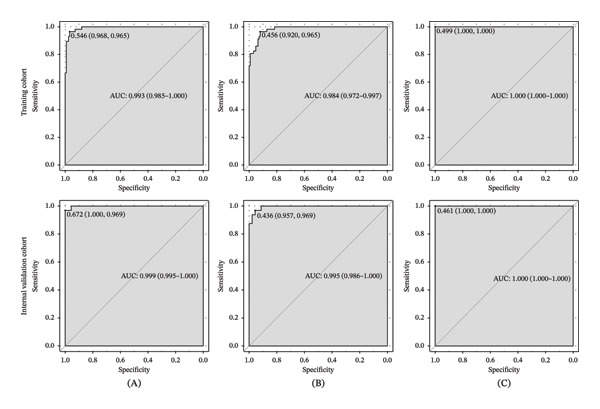
Receiver operating characteristic curves for predicting the probability of gout relief by using multivariable regression model (A), generalized Additive Model (B), and the XGBoost model (C).

In the multivariable logistic regression model, both URIC (OR [95 CI%]: 0.97 [0.95–0.99], *p* = 0.002), FAR (OR [95 CI%]: 0.59 [0.36–0.96], *p* = 0.035), VAS score (OR [95 CI%]: 0.005 [0‐0.076], *p* < 0.001), and the gout frequencies during 12 months before admission (OR [95 CI%]: 0.009 [0‐0.49], *p* = 0.021) were independently associated with gout relief (Table 2). The VIF of each variable were 1.0, 1.7, 3.2, 2.3, 2.5, and 1.3 for male, URIC, FAR, FPR, VAS score at admission, and gout frequencies during 12 months before admission (both < 5), implying that multicollinearity is not a concern in this model. Accordingly, a prediction model and nomogram were established, which demonstrated a good accuracy in estimating the probability of gout relief, with an AUC of 0.993 (95% CI: 0.985–1.00) in the training cohort (Figures 3 and 4). The AUCs were all higher than 0.98 in the multivariable logistic regression model, GAM, and the XGBoost model (both training cohort and internal validation cohort) (Figure 4). The impact of the input features on predictions in the XGBoost analysis is shown in Figure S2. In addition, the calibration curves were close to the ideal diagonal line in the three models (Figure S3).

**TABLE 2:** Multivariable logistic regression model in predicting gout relief.

	OR	95% CI	*p*
Male	1.04	0.95–1.20	0.487
URIC	0.97	0.95–0.99	0.002
FAR	0.59	0.36–0.96	0.035
FPR	0.67	0.14–3.09	0.605
Gout attack ≥ 3 times before admission	0.009	0–0.49	0.021
VAS			
0	Reference		
≥ 1	0.005	0–0.076	< 0.001

*Note:* PA denotes prealbumin, ALB denotes albumin, PA denotes prealbumin, and URIC denotes uric acid.

Abbreviations: VAS, visual analog scale; WBC, white blood cell; FAR, FBG/ALB ratio; FPR, FBG/PA ratio; FBG, fast blood glucose.

The age‐stratified and interaction analysis results showed that higher FAR was associated a lower gout relief rate among different age stratum, and the *p* for interaction was 0.246 (Table S1).

### 3.3. The Important Role of FAR in Predicting Gout Relief

**FIGURE 5:**
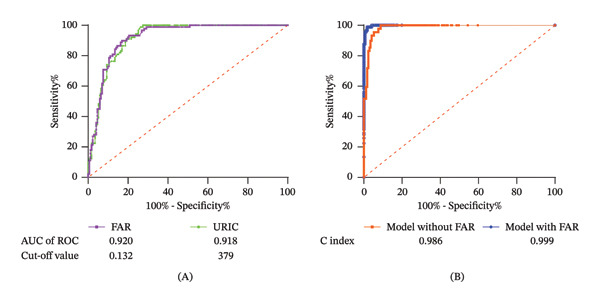
Receiver operating curves of PAR and URIC (A) and the model with FAR or without FAR (B) in predicting gout relief. FAR denotes FBG/ALB ratio, F FBG denotes fast blood glucose, ALB denotes albumin, and URIC denotes uric acid.

The ROC curves of FAR, URIC, and models with FAR or without FAR in predicting gout relief were shown in Figure 5. The AUC of FAR and URIC were 0.920 and 0.918, respectively. The predictive performance of FAR was not inferior to URIC (DeLong test *p* > 0.05). Compared with the prediction model without FAR, the model with FAR had a significantly higher C index (0.999 versus 0.986, DeLong test *p* = 0.016).

## 4. Discussion

In this study, it was found that a lower FAR at admission was associated with the increased probability of gout relief. The predictive performance of FAR in predicting gout relief was not inferior to the conventional URIC level at admission. The predictive performance of the model with FAR was significantly better than that without FAR, as reflected by a higher C index, implying the important role of FAR in predicting gout relief. Finally, we generated a novel and effective prediction model in predicting gout relief, which may provide a framework for applying this instrument in future clinical practice and research.

The association between a lower FAR and increased probability of gout relief may be explained by the complex interplay of metabolic and inflammatory processes in gout pathophysiology. FAR, as a composite marker combining FBG and ALB, potentially reflects glycemic control and nutritional‐inflammatory status, respectively [19, 20]. Elevated FBG levels have been linked to increased URIC production and decreased renal URIC excretion, exacerbating hyperuricemia [21]. Conversely, ALB, an important anti‐inflammatory protein, may play a protective role in gout by modulating the inflammatory response and oxidative stress [22]. Additionally, recent studies have suggested that ALB may interact with monosodium urate crystals, influencing their formation and dissolution [23]. A higher ALB concentration might facilitate the clearance of urate deposits, promoting gout relief. This multifaceted interaction between glucose metabolism, inflammation, and urate acid provides a plausible mechanistic basis for the observed predictive value of FAR in gout relief. However, further research is needed to elucidate the exact molecular pathways through which FAR influences gout outcomes.

The predictive performance of FAR for gout relief was comparable to that of conventional URIC levels. This finding is particularly significant, as URIC has long been considered the gold standard for gout diagnosis and monitoring [24]. Our findings suggest that FAR may offer additional prognostic value, potentially complementing URIC in clinical decision‐making. This is consistent with recent studies emphasizing the importance of considering multiple biomarkers for more accurate disease assessment in gout [25]. Notably, the inclusion of FAR significantly improved the predictive performance of our model, as evidenced by a higher C index. This improvement underscores the potential of FAR as a valuable prognostic tool in gout management. Similar enhancements in predictive models have been observed in other inflammatory conditions when incorporating novel biomarker ratios [26–28], supporting the broader applicability of our approach.

Despite the promising results, our study has several limitations that should be addressed. First, this was an exploratory single‐center study with a relatively small sample. Though we have performed internal validation to avoid overfitting, the lack of external validation may limit the generalizability of our findings to broader populations. Thus, the results of this study should be interpreted with caution. Future multicenter studies with larger cohorts are necessary to validate our results. Second, our study was retrospective in nature, which may introduce potential biases in data collection and analysis. While we controlled for several confounding factors, there may be additional unmeasured variables like the use of medications that could influence gout relief. Prospective trials that include treatment medicines are required to prove the predictive efficacy of FAR and our proposed model in real‐time clinical situations. Finally, our study focused on short‐term outcomes, and long‐term follow‐up studies are required to assess the sustained predictive value of FAR in gout management. Despite these limitations, our findings provide a strong foundation for future research and potential clinical applications in gout prognostication.

In conclusion, FAR at admission could be a novel and effective predictor for gout relief. The integration of FAR into a comprehensive prediction model represents a significant step forward in gout prognostication. These findings provide a framework for future clinical practice and research, potentially leading to more personalized and effective gout management strategies.

## Author Contributions

Wen Dai: original draft, conceptualization, data curation, software, validation, and writing–review and editing. Zebo Hu: data curation and writing–review and editing. Wenjian Mao: formal analysis, visualization, and writing–review and editing. Jia Wu: conceptualization, funding acquisition, supervision, resources, and writing–review and editing. Jun Hong: conceptualization, supervision, resources, writing–review and editing, and project administration.

## Funding

The authors have nothing to report.

## Disclosure

All authors have approved the final version of the manuscript.

## Conflicts of Interest

The authors declare no conflicts of interest.

## Supporting Information

Additional supporting information can be found online in the Supporting Information section.

## Supporting information


**Supporting Information** Figure S1. Least absolute shrinkage and selector operation (LASSO) analysis to filter out redundant factors. Figure S2. The impact of the input features on predictions in the XGBoost analysis. Figure S3. Calibrations curves for predicting the probability of gout relief using multivariable regression model (A), generalized additive model (B), and the XGboost model (C). Table S1. The association between FAR and gout relief among patients > 50 years old or ≤ 50 years old.

## Data Availability

Data can be accessed with the approval of the authors. Request for data can be made to the corresponding author and will be discussed during a meeting.
