# Supplementary material for: The Role of Fast Blood Glucose to Albumin Ratio in Predicting Gout Relief Among Patients With Acute Gout Attack
Source: Int J Endocrinol. 2026 Jul 24;2026:2656529. doi: 10.1155/ije/2656529 (PMC13400835; doi:10.1155/ije/2656529)
Supplement: Supplementary file 1 — Supporting Information Figure S1. Least absolute shrinkage and selector operation (LASSO) analysis to filter out redundant factors. Figure S2. The impact of the input features on predictions in the XGBoost analysis. Figure S3. Calibrations curves for predicting the probability of gout relief using multivariable regression model (A), generalized additive model (B), and the XGboost model (C). Table S1. The association between FAR and gout relief among patients > 50 years old or ≤ 50 years old. [file IJE-2026-2656529-s001.docx]

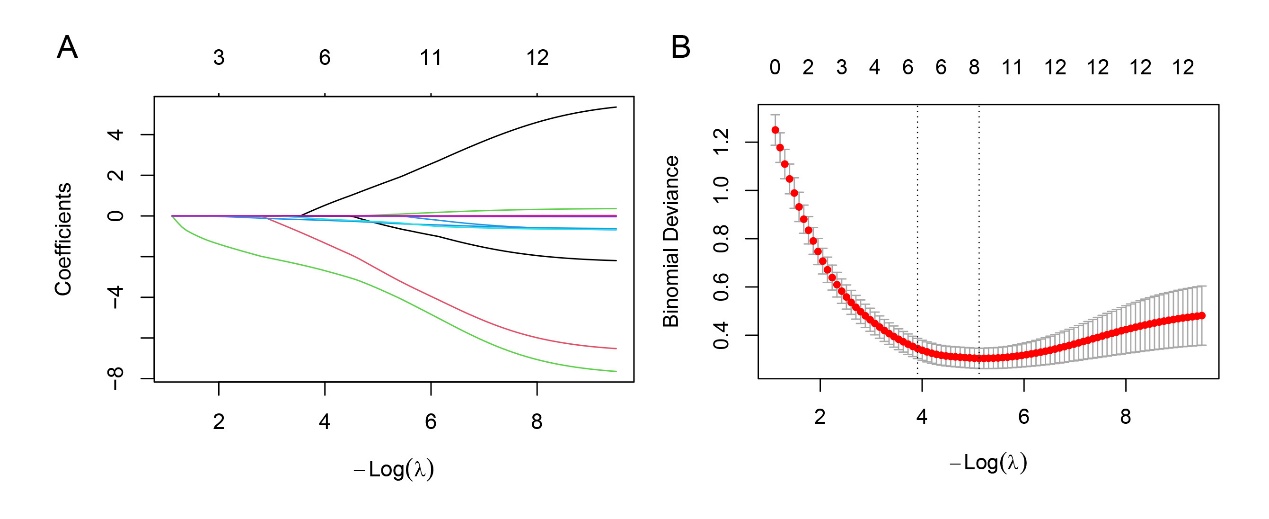


Figure S1. Least absolute shrinkage and selector operation (LASSO) analysis to filter out redundant factors.


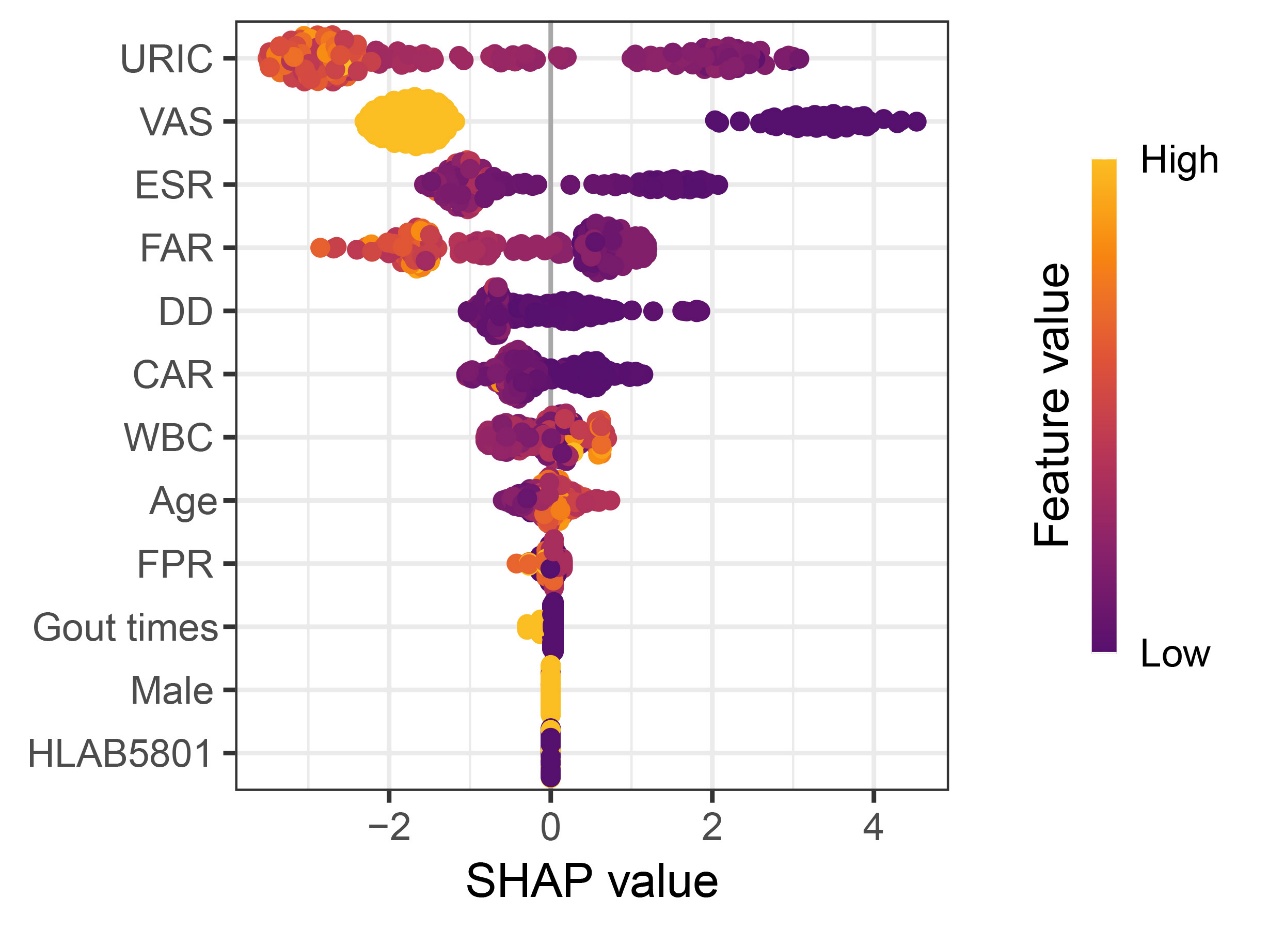


Figure S2. The impact of the input features on predictions in the XGBoost analysis. The Summary plot of the SHAP values for the top 12 significant features of each sample. The relationship between each feature and gout relief, with the colors reflecting the magnitude of each feature value (high in yellow, low in purple), with the points to the left of the central axis representing features that move the prediction towards gout non relief and the points to the right representing features that drive the prediction in gout relief direction.


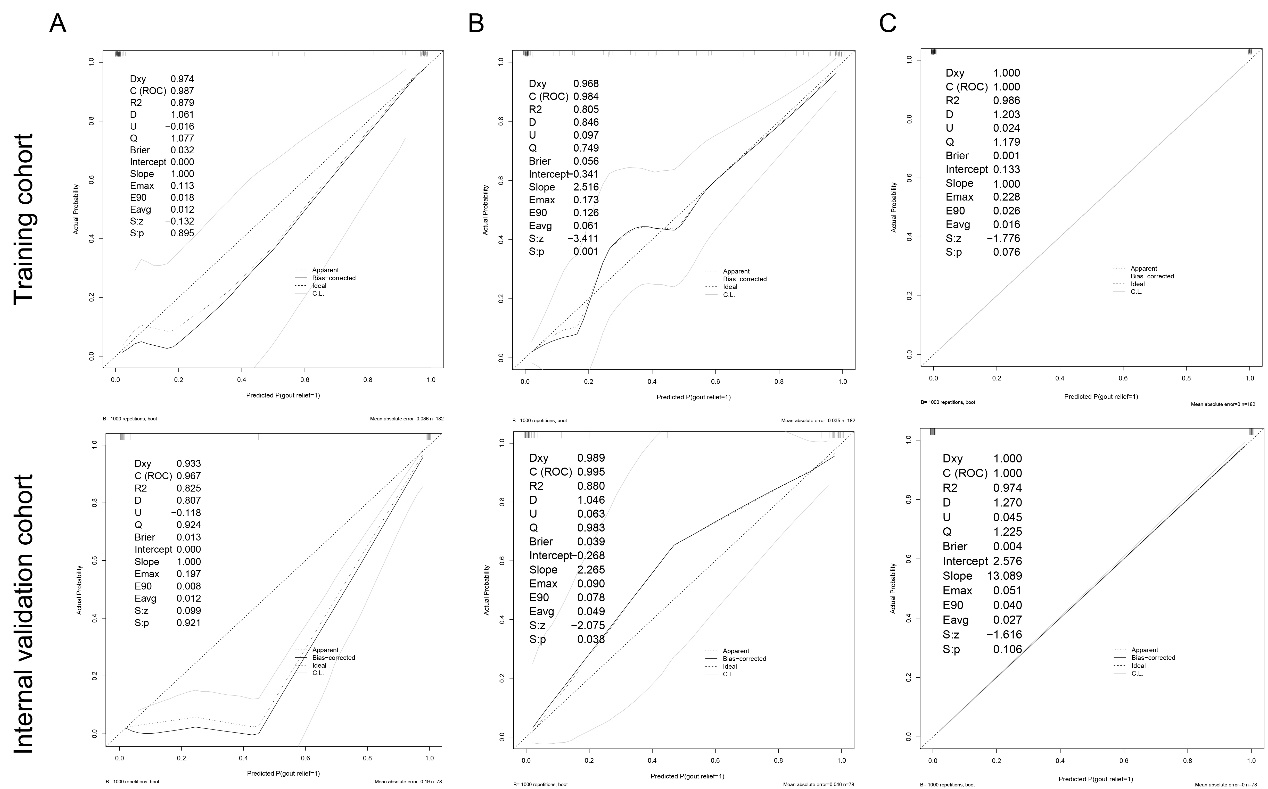


Figure S3. Calibrations curves for predicting the probability of gout relief using multivariable regression model (A), generalized Additive Model (B), and the XGboost model (C).

Table S1. The association between FAR and gout relief among patients >50 years old or ≤ 50 years old.

| Subgroup | OR | 95%CI | p | p for interaction |
| --- | --- | --- | --- | --- |
| Age |  |  |  | 0.246 |
| >50 | 0.43 | 0.31-0.61 | <0.001 |  |
| ≤50 | 0.55 | 0.44-0.70 | <0.001 |  |
